# Digital Economy Development, Industrial Structure Upgrading and Green Total Factor Productivity: Empirical Evidence from China’s Cities

**DOI:** 10.3390/ijerph19042414

**Published:** 2022-02-19

**Authors:** Yang Liu, Yanlin Yang, Huihui Li, Kaiyang Zhong

**Affiliations:** 1 Economics and Management School, Wuhan University, Wuhan 430072, China; 2 Center for Economic Development Research and Center of Population, Resource & Environmental, Economics Research, Wuhan University, Wuhan 430072, China; 00006652@whu.edu.cn; 3 Guangdong Rural Credit Union, Guangzhou 510627, China; lhhgdrcu@163.com; 4 School of Economic Information Engineering, Southwestern University of Finance and Economics, Chengdu 611130, China

**Keywords:** digital economy, green total factor productivity, industrial structure

## Abstract

The digital economy is an important engine to promote sustainable economic growth. Exploring the mechanism by which the digital economy promotes economic development, industrial upgrading and environmental improvement is an issue worth studying. This paper takes China as an example for study and uses the data of 286 cities from 2011 to 2019. In the empirical analysis, the direction distance function (DDF) and the Global Malmquist-Luenberger (GML) productivity index methods are used to measure the green total factor productivity (GTFP), while Tobit, quantile regression, impulse response function and intermediary effect models are used to study the relationship among digital economy development, industrial structure upgrading and GTFP. The results show that: (1) The digital economy can significantly improve China’s GTFP; however, there are clear regional differences. (2) The higher the GTFP, the greater the promotion effect of the digital economy on the city’s GTFP. (3) From a dynamic long-term perspective, the digital economy has indeed positively promoted China’s GTFP. (4) The upgrading of industrial structures is an intermediary transmission mechanism for the digital economy to promote GTFP. This paper provides a good reference for driving green economic growth and promoting the environment.

## 1. Introduction

Currently, promoting green development is the most effective way to prevent the outbreak and spread of unknown human infectious diseases from the source [1]. The COVID-19 pandemic further stimulated the public’s demand for changing the extensive development mode and pushing green development. 

In this way, as a booster of the high-quality development of the economy [2], the digital economy (according to the “G20 Digital Economy Development and Cooperation Initiative” released at the G20 Summit in 2016, the digital economy refers to a series of economic activities with the use of digital knowledge and information as key production factors, modern information network as an important carrier and the effective use of information and communication technology as an important driving force for efficiency improvement and economic structure optimization) has extended the industrial chain, spawned a series of new industries and upgraded (industrial upgrading is defined as the process that nations, firms and workers, as economic actors, move from low-value to relatively high-value activities in global production networks [3]) traditional industries. 

How does the digital economy affect industrial greening and green production? This is a cutting-edge problem worthy of research. China is a good sample for our study. First, the scale of China’s digital economy ranks among the top in the world. By the end of 2020, the scale of global digital economy had reached 32.6 trillion US dollars, and it accounted for 43.7% of the GDP (the data comes from the “White Paper on Global Digital Economy” released by China Academy of Information and Communications). China’s digital economy is 5.4 trillion US dollars, second only to the United States (the same data source as above). 

Second, China used to promote economic growth at the expense of the environment, resulting in excess pollution. At present, China has paid great attention to improving the environment. At the 76th United Nations General Assembly in 2021, China solemnly pledged to the world that it will strive to achieve a carbon peak by 2030 and achieve carbon neutrality by 2060. Achieving carbon peak and carbon neutrality is a tough battle, while improving GTFP and accelerating economic green transformation are the fundamental ways.

The main contributions of this paper are that we established an analytical framework for the impact of digital economy on green production and explored the mechanism by which digital economy promotes economic development, industrial upgrading and environmental improvements. In empirical analysis, we made the following choices: (1) GTFP is used to represent the development level of China’s green economy. The direction distance function (DDF) and Global Malmquist-Luenberger (GML) productivity index methods are used to measure GTFP. (2) Tobit, quantile regression, impulse response function and intermediary effect models are used to study the relationship among digital economy development, industrial structure upgrading and GTFP.

## 2. Literature Review

The concept of the digital economy first appeared in the book “The Digital Economy: Promise and Peril in the Age of Networked Intelligence” [4] written by Don Tapscott in 1996 and then formally put forward in the report “The Emerging Digital Economy” published by the US Department of Commerce in 1998. The global digital economy has shown a rapid growth trend in the development process of more than 20 years and has become a new engine to promote the global economic recovery. 

The digital economy has effectively stabilized the downward trend of China’s economy under the impact of the pandemic. At the same time, during the pandemic, the digital economy played a fundamental role in supporting the fight against the COVID-19 pandemic, resuming work, resuming production and resuming school [1]. Compared with the traditional offline economy, which relies on physical space, the digital economy has shown wide application prospects and great growth potential due to the advantages of network and data space [5,6]. 

The digital economy has become the most dynamic, innovative and radiating economic form, and it has become one of the core growth poles of the national economy [7]. It is foreseeable that, in the post-pandemic era, the high-quality development of China’s economy will urgently need the guidance of the digital economy.

The essence of the digital economy is a special economic form that trades goods and services through virtualization. Its development is closely related to the information and communication technology industry, and it accelerates penetration and changes the operation mode of related industries [8,9,10]. The digital economy is a new economic and social form, and data has become a new production factor in addition to capital, labor and land [11]. The advantages of the digital economy are convenient information acquisition, rich interactions and low information and interaction costs [12,13,14]. The international research on digital economy has gone through the exploration process from informatization and the internet to digital economy. 

Roller and Waverman (2001) indicated that the popularization of information and communication equipment can significantly promote regional economic growth [15]. Antonelli (2003) proposed that the introduction of information and communication technology (ICT) has made it possible to greatly improve the level of total factor productivity in the United States [16]. Oliner et al. (2008) used US industry data and found that information technology played an important role in the economic recovery from 1995 to 2000 [17]. Greenstein and McDevitt (2011) estimated the contribution of broadband internet to US GDP, and the results showed that the new revenue generated by broadband internet accounted for 40–50% of GDP [18]. Research by Jiménez et al. (2014) showed that, in Mexico, internet access had a positive impact on economic growth [19].

With the development of digital technologies, such as big data and artificial intelligence, the focus of academic circles has gradually shifted to the digital economy. Studies by Ivus and Boland (2015) and Jorgenson (2016) showed that the digital economy can accelerate a country’s economic growth [20,21]. Acemoglu and Restrepo (2018) improved the neoclassical model by adding the hypothesis that machine intelligence and human labor complement each other and found that the application of machine intelligence can increase the economic growth rate by an order of magnitude or more [22]. 

Graetz and Michaels (2018) used the data of industrial robots in 17 countries from 1993 to 2007 to find that industrial robots can promote the improvement of TFP [23]. Sutherland (2018) proposed that the contribution rate of digital economy to GDP is gradually increasing and that it is the most active economic development field in recent years [24]. Chakpitak et al. (2018) studied how the growth of digital technology affected the Thai economy. The results showed that digital technology had little positive contributions to the Thai economy and that digital technology had not been used to the maximum in Thailand and there was still room for improvement [25]. Pan et al. (2022) tested the innovation driving effect of digital economy on China’s TFP, and the research results showed that the digital economy was the innovation driving force for the extensive and sustainable development of China’s TFP [26].

Li et al. (2020) suggested that GTFP is not only an inevitable choice to continuously increase the quality of China’s economy but also a booming demand to promote global development [27]. Zhou and Wang (2021) indicated that GTFP growth has become a key measurement indicator for the development and transformation of green economy, and it also reflects the essence of high-quality development [28]. Buhkt and Heeks (2017) proposed that the development of digital economy will accelerate the formation of a new business model with green characteristics covering the platform economy and sharing economy [29]. 

Guo Han (2020), a Chinese scholar, also suggested that fully integrating the digital economy into the real economy will help enterprises to improve production mode, increase industrial productivity and accelerate the green transformation of the real economy [30]. With the development of digital economy, we can build a green three-way interactive bridge between government, enterprises and the public by building a digital platform to ensure the quality of ecological environment [31]. 

The digital economy can improve the ecological efficiency of cities by constructing a feedback mechanism of ecological protection and spreading the positive concept of green life [32]. Li et al. (2020) studied the impact of the development of the internet on China’s GTFP and proposed that the development of the internet has had a significant positive impact on GTFP through the integration of resources and the application of energy-saving technologies [27].

The existing literature has important reference significance for the further development of this study; however, there are also the following problems to be studied, which are correspondingly expanded in this paper: 

(1) Most existing literature discusses the relationship between digital economy and national economic development from the provincial level. This paper makes a comprehensive study of digital economy and China’s GTFP from the city level, and we discuss the relationship between them on a more subtle scale. 

(2) Existing literature mainly studies the influence of the internet development and digital economy on TFP; however, few studies discuss the influence of digital economy on GTFP. In this paper, the GTFP is used to represent the development level of China’s green economy. When calculating the GTFP, the factors of energy input and environmental pollution are considered to study the influence of digital economy on the GTFP. 

(3) In terms of research methods, most literature uses the Granger causality test, OLS regression and threshold regression to study the influence of digital economy on economic development. In this paper, the Tobit model is used to study the overall influence of digital economy on China’s GTFP, the quantile regression model is used to study the conditional distribution of influence characteristic of digital economy on the GTFP, and an impulse response function is used to study the dynamic influence of digital economy on GTFP. 

(4) Most of the existing literature studies the direct mechanism of the digital economy on national economic development and seldom pays attention to the important intermediary mechanism of industrial structure. This article uses the key intermediary variable of industrial structure to demonstrate from both theoretical and empirical aspects that the digital economy can realize the green transformation and sustainable development of the economy by optimizing and upgrading the industrial structure.

## 3. Theoretical Analysis

As the core force of the new round of industrial transformation, the digital economy takes data as the key factor of production, which has the innate characteristics of high technology, high growth, platform-based, cross-time and space communication, data creation and data sharing, which effectively breaks the contradiction between supply and demand of city’s green economy development factors. The digital economy not only has a direct impact on the development of city’s green economy through its own characteristics but also indirectly affects the development of city’s green economy by optimizing and upgrading the industrial structure of cities. 

No matter whether it is German Industry 4.0, American Industrial internet or “Made in China 2025”, big data is an extremely important strategic resource, known as “oil in the new era”, which provides new kinetic energy for the transformation and upgrading, quality improvement and efficiency improvement and sustainable development of manufacturing industry. Industrial structure is an important link between resources, environment and economic development [33]. Industrial structure plays an important role in economic growth [34,35,36]. 

The traditional school of industrial structure, represented by the Petty-Clark theorem, believes that industrial structure adjustment can bring “structural dividends” [37]. The theory of the “structural dividend hypothesis” shows that the optimization of industrial structure is helpful to guide the factors of production to the industrial sectors with high productivity, and the resulting “structural dividends” improve the productivity level of the whole society, and changes of industrial structure will affect the pollution level [38] so as to promote the sustainable development of the economy [39]. 

Unreasonable industrial structure will distort the allocation of factors, increase the waste of resources and lead to low efficiency of economic development [40]. This study discusses that the digital economy can promote the optimization and upgrading of industrial structure from three aspects: green and low-carbon development, value distribution transfer and the demand changes forced. According to the “structural dividend hypothesis”, the development level of city’s green economy will be improved. 

The digital economy empowers the transformation and upgrading of traditional industries; promotes the green development, intensive development and environmental development of industries; accelerates the conversion and upgrading of new and old kinetic energy; and takes the road of sustainable development. The development level of city’s green economy has been improved. 

Take the technology of the Internet of Things as an example. The Internet of Things can effectively reduce the energy consumption and pollutant emissions of energy-intensive manufacturing industries by perceiving and collecting data from the manufacturing process of energy-intensive industries with the help of big data analysis technology, which leads the way for the green development of manufacturing industries and the formation of ecological industrial structure [41]. The World Economic Forum (WEF) estimates that, by 2030, expanding the use of digital technologies could reduce global carbon emissions by at least 15%. Data from the “Exponential Climate Action Roadmap” (2018) also shows that digitalization could reduce global carbon emissions by 15%. 

In addition to remarkable achievements in reducing carbon emissions, digital technology has great potential in many other aspects of environmental protection. According to the GeSI data (2015), by 2030, the application of digital technology can increase the per unit area yield of crops by 30%, which means that the annual yield per hectare will be increased by nearly 900 kg of grain, and more than 300 trillion liters of water and 25 billion barrels of oil will be saved every year [42].

The digital economy can help to transform the organizational form of manufacturing industry chain and reshape the value distribution form of manufacturing industry chain. The value distribution in the manufacturing industry chain is that the highest is in the upstream R&D design and downstream marketing and service links, and the manufacturing link in the middle of the industry chain is the lowest, showing a “smile curve” form. The application of digital technologies, such as the Internet of Things, artificial intelligence and cloud computing has comprehensively improved the production efficiency and value creation space of the manufacturing industry chain. 

The assembly manufacturing link in the middle part of the industrial chain has the characteristics of standardization in its production process or mode, and the productivity increase range and value increase are larger than the R&D design and marketing service links at both ends of the industrial chain. At the same time, based on the function of resource allocation optimization and function integration of digital platform, the competitive enterprises in each link of the industrial chain will tend to form a community of interests with synergistic effect around the core enterprises or digital economic platform in the link, and the strong competitive relationship will turn to a new type of competition-cooperation relationship, which will promote the upgrading of the industrial value chain to a certain extent.

The rapid rise of digital economic platform has opened up a new market space for the industrialization of digital technology, spawned a number of new technologies and new business modes and then triggered the change of consumer demand. Based on big data analysis technology, the digital economic platform improves the matching efficiency of information, reshapes the matching mode of supply and demand information in the terminal of industrial chain and stimulates new consumption potential. 

Consumers’ demand information for products is fed back from the downstream to the upstream of the industrial chain and integrated into all links of the industrial chain, such as R&D design, manufacturing, marketing and service, which effectively forms the traction effect of the demand side on the supply side and promotes the transformation from production manufacturing to service manufacturing. 

Under the platform business mode of the digital economy, the personalized and diversified demands of consumers are amplified, and the demand for small quantities of personalized products is improved, which proves the “long tail effect” of the digital economy. Under the force of changes in demand, the transformation and upgrading of the production organization mode of the manufacturing industry and the restructuring of the industrial chain organization structure on the supply side have also become inevitable.

## 4. Materials and Methods

### 4.1. Data Source and Sample Selection

The sample data for this paper comes from the “China City Statistical Yearbook”, “China Statistical Yearbook”, “Statistical Yearbook” of China’s provinces (autonomous regions and municipalities), “Statistical Communique of National Economic and Social Development” of Chinese cities and the Enterprise Big Data Research Center of Peking University. For partially missing data, linear interpolation is used to supplement. 

This paper selected the data of 286 prefecture-level and above cities (obtained according to the city classification in the “China City Statistical Yearbook”) in China from 2011 to 2019 as research samples. In order to reduce the magnitude difference of variables, prevent the heteroscedasticity problem from causing estimation bias and, at the same time, clarify the coefficient of elasticity of change between variables, each variable in the empirical regression of this study is presented in logarithmic form.

#### 4.1.1. Relevant Indicators for GTFP Measurement

The development of green economy refers to improving the level of input and output on the basis of minimizing environmental damage and loss of natural resources and promoting sustainable economic development and coordinated development of society and ecology [33]. GTFP incorporates resources and environmental factors into the productivity analysis framework for research, which is in line with the concept of green development in the new era [43,44,45,46,47]. Therefore, GTFP can better measure the development level of green economy.

In this paper, the following input, expected output and unexpected output indicators are used to measure the GTFP of each city by using directional distance function (DDF) and Global Malmquist-Luenberger (GML) index. Input indicators: 

(1) Capital input: represented by capital stock, using the perpetual inventory method to calculate the capital stock of each city at constant prices after estimating a base year by referring to Zhang Jun et al. (2004) [48]. The formula is: $K_{it}=K_{it-1}(1-\delta )+I_{it}/P_{it}$. Among them, $I_{it}$ is the total amount of fixed assets formed in the current year, δ is the depreciation rate (here, at 9.6%), and $P_{it}$ is the fixed assets investment price index. In order to eliminate the influence of inflation, the fixed assets investment index of each province in the corresponding year is used to convert the fixed assets investment of each city into the fixed assets investment based on 2015. The capital stock of the base period refers to the method of Young (2003) [49], Zhang Jun et al. (2004) [48] to obtain the capital stock in 2015—that is, the amount of fixed assets formed by each city in 2015 is divided by 10% as the initial capital stock of the city. 

(2) Labor input: Measured by the employees of the whole society—that is, the sum of employees in urban units, private and individual employees. 

(3) Energy input: Use the electricity consumption of the whole society to characterize [50]. Lin Boqiang (2003) [51] indicated that electricity consumption and energy consumption have a high correlation and accuracy; therefore, electricity consumption is used as an indicator to measure energy input. 

The expected output index is characterized by the real GDP and adjusted to a constant price in 2011 according to the GDP deflator. Undesired output indicators are measured by industrial wastewater, industrial sulfur dioxide and industrial smoke (dust) emissions.

Based on the above input and output indicators, the GTFP of 286 cities in China from 2011 to 2019 was calculated by using the MaxDEA8.21 software. The GML index reflects the change rate of GTFP in the current year relative to the previous year; therefore, the GTFP of the base period in 2011 is set to 1, and then we multiply this with GML index of each year in turn to obtain the GTFP of each year.

#### 4.1.2. Digital Economy Indicators

Referring to the paper by Zhao Tao (2020) [52], this paper uses the internet penetration rate, related employees, related output, mobile phone penetration rate and digital inclusive finance to measure the development level of digital economy. The specific indicators are: the number of internet broadband access users per 100 people, the proportion of computer service and software employees, the per capita telecommunication service, the per capita postal service, the number of mobile phone users per 100 people and the development of digital inclusive finance. 

Among these, the development of digital inclusive finance is characterized by the digital inclusive finance index jointly compiled by the Digital Finance Research Center of Peking University and Ant Financial Group [53]. Compared with the digital economy index constructed by Zhao Tao (2020) [52], this paper adds an index of postal service per capita to represent the online shopping situation of Chinese netizens, which can reflect the level of development of e-commerce. In addition, for some data with large fluctuations, this paper smooths the data according to the average annual growth rate of the indicators. Finally, the topsis entropy weight method is used to calculate the digital economy development index at the city level in China.

#### 4.1.3. Intermediary Variables

Taking the upgrading of industrial structure as the intermediary variable, this paper draws on the method of Fu Linghui (2010) to construct the advanced index of industrial structure to represent the degree of industrial structure upgrading [54]. First, we construct a set of three-dimensional vectors $X_{1}=(1,0,0)$, $X_{2}=(0,1,0)$, $X_{3}=(0,0,1)$, which are arranged from low to high industrial levels. Then, a set of three-dimensional vectors $X_{0}=(x_{1,0},x_{2,0},x_{3,0})$ of the added value of tertiary industries as a proportion of GDP is constructed. Then, we calculate its angles $\theta_{1}$, $\theta_{2}$ and $\theta_{3}$ with $X_{1}$, $X_{2}$ and $X_{3}$.

(1) $$\theta_{j}=arccos\left(\frac{\sum_{i=1}^{3}\left(x_{i,j}x_{i,0}\right)}{\left(\sum_{i=1}^{3}{\left(x_{i,j}^{2}\right)}^{1/2}\sum_{i=1}^{3}{\left(x_{i,0}^{2}\right)}^{1/2}\right)}\right)$$

Among them, j=1,2,3. The industrial structure advanced index is finally obtained:

(2) $$W=\overset{3}{\underset{k=1}{\sum }}\overset{k}{\underset{j=1}{\sum }}\theta_{j}$$

#### 4.1.4. Control Variables

This paper selected environmental regulation, innovation and entrepreneurship, marketization level, foreign direct investment, human capital, financial development, government financial expenditure on science and technology and the number of enterprises as control variables. 

Among them, environmental regulation is measured by the ratio of the frequency of environmental words to the frequency of words in the government work reports of prefecture-level cities. 

Referring to the practice of Zhang Jianpeng and Chen Shiyi (2021) [55], this paper selected 27 environmental words that can fully reflect the government’s emphasis on environmental protection from three aspects: environmental protection objectives, environmental factors and pollution and environmental protection measures. 

Based on this word set (the word set refers to the following 27 environmental protection words: environmental protection, abbreviation of environmental protection, green, clean, low carbon, blue sky, green water, green mountains, ecology, air, climate, pollution, sulfur dioxide, chemical oxygen demand, fog and haze, particulate matter, carbon dioxide, energy consumption, loose coal, burning coal, drain contamination, secretly discharge, tail gas, energy conservation, emission reduction, desulfurisation and denitration), R software [56] was used to develop text statistics and analysis on the government work reports of prefecture-level cities. 

The Innovation and Entrepreneurship Index comes from the Enterprise Big Data Research Center of Peking University. The index is composed of several dimensions: new enterprises, attracting external investment, attracting venture capital, the number of patent authorizations and the number of trademark registrations. The level of marketization is expressed by the proportion of urban private and individual employees in urban unit employees. Foreign direct investment is measured by the proportion of foreign capital actually used in each city to GDP in that year. 

Human capital investment is expressed by the proportion of education expenditure to public financial expenditure. Financial development is measured by the proportion of various RMB loan balance of financial institutions to GDP at the end of the year. Government expenditure on science and technology is characterized by the proportion of government expenditure on science and technology to public financial expenditure. The number of enterprises is measured by the number of industrial enterprises above designated size. Descriptive statistics of variables are shown in Table 1.

### 4.2. Research Strategy

We mainly studied the influence of digital economy on the development of city’s GTFP and focused on the intermediary mechanism of industrial structure upgrading. First, this paper uses the DDF and the GML productivity index methods to measure city’s GTFP. Secondly, the Tobit model is used to carry out the benchmark regression of digital economy to city’s GTFP. Then, the quantile regression model is used to explore the influence of the digital economy on the overall conditional distribution of city’s GTFP. 

In addition, impulse response function is used to study the dynamic influence of digital economy on city’s GTFP. Aiming at the endogenous problem that may appear in this paper, the IV-Tobit method is used to deal with it. Finally, through the intermediary effect model, the theoretical hypothesis that the digital economy can realize green economy development by promoting industrial structure upgrading is verified.

### 4.3. Research Methods

#### 4.3.1. Measuring Method of GTFP

Referring to the ideas of Fare et al. (2007) [57], a production possibility set of environ mental technology including expected output and unexpected output is constructed first. This paper regards each city as a production decision-making unit, assuming that each decision-making unit needs to input *N* kinds of production factors $x=(x_{1},x_{2},\cdot \cdot \cdot ,x_{N})\in R_{+}^{N}$ in the production process. This produces *M* kinds of expected outputs $y=(y_{1},y_{2},\cdot \cdot \cdot ,y_{M})\in R_{+}^{M}$ and *I* kinds of unexpected outputs $b=(b_{1},b_{2},\cdot \cdot \cdot ,b_{I})\in R_{+}^{I}$. 

It is further assumed that the production possibility set satisfies the axiom of zero combination, the expected output and input elements satisfy the strong disposability and the unexpected output also satisfies the axiom of weak disposability and thus that the production possibility set of environmental technology is expressed as:

(3) $$P\left(X\right)=\left\{\left(x,y,b\right):\overset{K}{\underset{k=1}{\sum }}z_{k}x_{kn}\leq x_{kn},\overset{K}{\underset{k=1}{\sum }}z_{k}y_{km}\geq y_{km},\overset{K}{\underset{k=1}{\sum }}z_{k}b_{ki}=b_{ki},z_{k}\geq 0;\forall k,n,m,i\right\}$$

In Formula (3), P(X) represents the production possibility set of environmental technology, $z_{k}$ represents the weight of each decision-making unit, if $\sum_{k=1}^{K}z_{k}=1$, which means that environmental technology is variable in scale return (VRS), and, if this constraint is removed, it means that environmental technology is constant in scale return (CRS).

Chung et al. (1997) [58] pioneered the use of pollutants as undesired output and used the directional distance function (DDF) and Malmquist-Luenberger (ML) index to measure the GTFP. In this paper, referring to the research of Chung et al. (1997) [58], the directional distance function is defined as:

(4) $$\vec{D}\left(x,y,b,\vec{g}\right)=max\left\{\beta :\left(x-\beta {\vec{g}}_{x},y+\beta {\vec{g}}_{y},b-\beta {\vec{g}}_{b}\right)\in P\left(X\right)\right\}$$

In Formula (4), $\vec{g}=({\vec{g}}_{x},{\vec{g}}_{y},{\vec{g}}_{b})$ is the directional vector of each input and output variable, which represents the increase or decrease of elements. β is the vector of inefficiency term, which represents the change of inefficiency.

With the directional distance function, the TFP index can be constructed. The ML index is expressed in the form of a geometric average, and it is not transitive in the process of analysis, which makes it difficult to observe the long-term change trend of productivity index. At the same time, the ML index also faces the problem that there is no feasible solution for linear programming when measuring the intertemporal direction distance function (Oh, 2010) [59]. 

The Global Malmquist-Luenberger (GML) index method can not only effectively compensate for the defects of the ML index but also reduce the possibility of inward shifting of the production frontier. Therefore, referring to the research of Oh (2010) [59], this paper defines an intertemporal GML index as follows:

(5) $$GML^{t,t+1}\left(x^{t},y^{t},b^{t},x^{t+1},y^{t+1},b^{t+1}\right)=\frac{1+D^{G}\left(x^{t},y^{t},b^{t}\right)}{1+D^{G}\left(x^{t+1},y^{t+1},b^{t+1}\right)}$$

In Formula (5), $D^{G}(x,y,b)$ is the global directional distance function defined under the global production possibility set $P^{G}(X)$. $GML^{t,t+1}$ represents the GTFP from the *t* period to the *t* + 1 period. When $GML^{t,t+1}$ > 1, it means that the GTFP increases. When $GML^{t,t+1}$ < 1, it indicates that the GTFP decreases. When $GML^{t,t+1}$ = 1, it means that the GTFP remains unchanged.

The DDF and the GML productivity index method belongs to data envelopment analysis (DEA) of nonparametric frontier analysis. Measuring TFP by DEA has the following advantages compared with the Growth Accounting Approach [60,61], which is widely used [62,63,64,65]. As we know, the Growth Accounting Approach is a parametric method, which is limited by the function form. Choosing different production function models will lead to different estimation results, which affects the generality of this method. No matter what kind of function there are assumptions, the simulated production conditions are far from the actual conditions. 

DEA uses linear programming methods to evaluate the relative effectiveness of comparable units [66]. In actual research of efficiency evaluations, scholars mostly use the DEA method, avoiding the estimation and inference of the specific form of the production function [67]. The DEA can deal with multi-inputs and multi-outputs, especially the unexpected outputs and can be used to measure the green total factor productivity considering the unexpected output of environmental pollution.

#### 4.3.2. Tobit Model

Considering that the value of GTFP is a non-negative truncated data, which belongs to the restricted dependent variable, if the ordinary least square method is used for regression, the estimated value of its parameters may be biased and inconsistent. In order to solve this problem, Tobin (1958) [68] first proposed the Tobit model and suggested that, for restricted explained variables, the Tobit model that follows the maximum likelihood estimation is a better choice. Based on the research of Wu Lei et al. (2020) [69], this paper uses the panel Tobit model of random effect.

(6) $$GTFP_{i,t}=\alpha_{0}+\alpha_{1}digital_{i,t}+\alpha_{2}Z_{i,t}+u_{i,t}+\varepsilon_{i,t}$$

In Formula (6), ${\mathrm{GTFP}}_{i,t}$ represents GTFP, *i* represents city, *t* represents time, $digital_{it}$ represents the level of digital economy development, regression coefficient $\alpha_{1}$ reflects the impact of digital economy development on GTFP, $Z_{it}$ represents control variable, $u_{i,t}$ represents individual error, and $\varepsilon_{i,t}$ represents random error.

#### 4.3.3. Quantile Regression Model

In this study, the quantile regression method is used to analyze the change of the influence of the digital economy on GTFP at different levels. The quantile regression method was originally proposed by Koenker and Bassett (1978) [70], which is a regression method to fit the linear function of the explanatory variable based on the conditional distribution of the explained variable. 

The estimation coefficient of quantile regression indicates the marginal effect of independent variable on dependent variable at a specific quantile, which can fully reflect the conditional distribution characteristics of dependent variable, especially the effective description of local information of distribution function, thus avoiding one-sided judgment of research problems based on “average” influence. In view of the above characteristics, quantile regression has become the best method to study the effect of differentiation and the influence characteristic of the entire conditional distribution.

Suppose the form of the distribution function of the random variable is as follows:

(7) F(y)=P(Y≤y)

Then the τ(0<τ<1) quantile function of *y* can be defined as:

(8) Q(τ)=inf{y:F(y)≥τ}

In Formula (8), τ represents the proportion of data below the regression line (plane) in the total data. In the distribution of y, the proportion of τ is less than Q(τ), while the proportion of (1-τ) is greater than Q(τ), and the distribution of y is divided into two parts by τ.

The probability function is defined as follows:

(9) $$\rho_{\tau }\left(\mu \right)=\{\begin{array}{c} \tau \mu {\;\mathrm{when}\;Y}_{i}\geq X_{i}^{\prime }\beta \\ \left(\tau -1\right)\mu {\;\mathrm{when}\;Y}_{i}\leq X_{i}^{\prime }\beta \end{array}$$

In Formula (9), μ is a parameter reflecting the probability density function, and $\rho_{\tau }(\mu )$ represents the probability density function relationship when the sample point of y is below and above the t quantile. Suppose the quantile regression model is:

(10) $${\hat{y}}_{Q}=\alpha_{Q}+\beta_{Q}x$$

The quantile regression of *y* is to find the minimum absolute deviation sum of *y* under Q quantile, and the expression is as follows:

(11) $$\underset{\beta }{min}\sum \left|y_{iQ}-\alpha_{Q}-\beta_{Q}x_{i}\right|*\rho_{iQ}$$

In the actual estimation process, it is generally assumed that μ = 1, then for any τ quantile regression, parameter estimation is to minimize the sum of squares of the absolute values of weighted errors, and the expression is as follows:

(12) $$\hat{\beta }\left(\tau \right)=argmin\underset{y_{i}\geq x_{i}^{\prime }\beta }{\sum }\tau \left|y_{i}-x_{i}^{\prime }\beta \right|+\underset{y_{i}<x_{i}^{\prime }\beta }{\sum }\left(1-\tau \right)\left|y_{i}-x_{i}^{\prime }\beta \right|$$

According to Formula (12), when τ takes different values in (0,1), different parameter estimates can be obtained.

#### 4.3.4. PVAR Model

The PVAR model, namely panel vector autoregressive model, was first proposed by Holtz-Eakin et al. (1988) [71]. In this model, all variables in the system are regarded as endogenous variables, and the lag terms of all variables are considered, which can reflect the relationship between variables more truly and effectively. PVAR model integrates the advantages of panel data model and VAR model, allowing individual effects among sample individuals and time effects on cross section. 

By increasing the number of cross sections, the observed values of samples are enlarged, and the restrictive requirements of VAR model on the length of time series are reduced, so that the influence of individual differences of sample units on model parameters can be better captured. The PVAR model can decompose the dynamic impact of each shock on the variables in the system through the impulse response function under the condition that other variables remain unchanged. The PVAR model has been continuously improved and matured by scholars, such as Mccuskey and Kao (1998) [72], Love and Zicchino (2006) [73]. Its basic form is as follows:

(13) $$y_{i,t}=\beta_{0}+\overset{p}{\underset{j=1}{\sum }}\beta_{j}y_{i,t-j}+f_{i}+e_{i}+\varepsilon_{i,t}$$

In Formula (13), $y_{i,t}$ is the k-dimensional endogenous variable column vector, *i* rep- resents the cross-section individual, *t* represents the time, *p* represents the model lag order, $\beta_{0}$ is the intercept term vector, $\beta_{j}$ is the parameter matrix of the lagged variable, $f_{i}$ is the individual effect column vector, $e_{i}$ is the time effect column vector, and $\varepsilon_{i,t}$ is the random disturbance term.

#### 4.3.5. Mediating Effect Model

In addition to the direct effect of the digital economy on GTFP, through the analysis of the theoretical mechanism in the previous section, the digital economy may also have an impact on GTFP by promoting the upgrading of industrial structure. In order to verify the theoretical mechanism, this study makes an empirical test according to the gradual causality method of mediation effect proposed by Baron and Kenny (1986) [74]. The specific test steps are as follows: On the basis of passing the test of the significance of in the Tobit regression model (6) of digital to GTFP, the Tobit regression model of digital to intermediary variable industrial structure (indus) and the Tobit regression model of digital and indus to GTFP are constructed, respectively. The specific setting form of the intermediary effect model is as follows:

(14) $$M_{i,t}=\gamma_{0}+\gamma_{1}digital_{i,t}+\gamma_{2}Z_{i,t}+u_{i,t}+\varepsilon_{i,t}$$

(15) $$GTFP_{i,t}=\eta_{0}+\eta_{1}digital_{i,t}+\eta_{2}M_{it}+\eta_{3}Z_{i,t}+u_{i,t}+\varepsilon_{i,t}$$

Among them, $M_{it}$ represents the industrial structure. If $\alpha_{1}$, $\gamma_{1}$ and $\eta_{2}$ are all significant and $\left|\eta_{1}\right|<\left|\alpha_{1}\right|$, then the mediating effect exists.

## 5. Results

### 5.1. Tobit and OLS Regression Results and Analysis

In this paper, Tobit and OLS regression models are used to preliminarily verify the influence of digital economy development on GTFP. The regression results are shown in Table 2. The Tobit regression results at the national level show that the estimated coefficient of the core explanatory variable digital economy (digital) is 0.3549, which is significant at the 1% level. 

This shows that the development of the digital economy can effectively promote the improvement of GTFP in Chinese cities. The Tobit regression results of the eastern and central regions show that the estimated coefficient of digital economy is 0.4194, which is higher than the national average level and significant at 1%. This shows that the development of digital economy in the eastern and central regions has greatly improved the level of GTFP in local cities. The Tobit regression results of the western region show that the estimated coefficient of digital economy is −0.1495, which is not significant. This indicates that the development of the digital economy in the western region has not played a role in improving the GTFP. 

The OLS regression results at the national level show that the estimated coefficient of digital economy is 0.2497, which is significant at the 1% level. The OLS regression results of the eastern and central regions show that the estimated coefficient of digital economy is 0.2940 and significant at 1%. The OLS regression results of the western region show that the estimated coefficient of digital economy is 0.1955, which is not significant. The economic implications of the OLS regression results are consistent with the Tobit regression results.

There is clear regional heterogeneity in the role of digital economy in GTFP. The possible reasons are as follows. The first is that the modern information network, as the carrier of the digital economy, determines the process of information collection, transmission and search. The level of industrial digitization and digital technology application in the eastern and central regions is much higher than that in the western region. The level of development of the digital economy is also relatively advanced with a larger scale and higher quality of data information. 

The second is that the level of economic development in the eastern and central regions is relatively high, the division of industry is more detailed and the degree of economic integration is higher, which enables the digital economy to effectively improve the GTFP of the regions. In contrast, the development level of digital economy in the western region is relatively low, and the quantity and quality of digital information need to be improved. 

The third is that the use of information technology requires a certain level of education, and residents in relatively backward areas have poor ability to use information technology (Bonfadelli, 2002) [75]. In addition, the capital accumulated in reality will be transformed into internet capital through internet access. Under the network effect, the problem of development inequality between regions and individuals will become more serious [76,77]. The research of Yang Wenpu (2021) [78] shows that there is a digital divide between regions in China, and there is an imbalance between regions in digital infrastructure construction. According to the research of Hawash and Lang (2020) [79], there is still a gap in the use of digital information technology as well as a capability gap caused by differences in users’ abilities and skills. 

As a result, the underdeveloped regions cannot fully enjoy the digital dividends, and the gap between them and the developed regions may become increasingly prominent in the process of economic growth.

### 5.2. Quantile Regression Results and Analysis

Given that the general panel regression model focuses on the influence of explanatory variables on the conditional expectation of explained variable, this is essentially a mean regression. This study uses quantile regression to study the influence of the digital economy on the overall conditional distribution of GTFP, which can better explore the differential influence of the digital economy on different levels of GTFP. Thus, the influence characteristic of digital economy on the whole conditional distribution of GTFP is obtained. 

**Figure 1:**
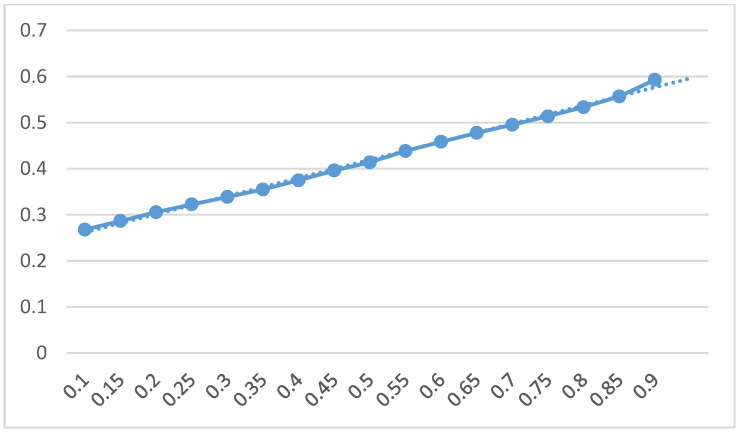
The regression coefficient diagram of the digital economy at each quantile.

In this study, quantile regression is performed every 5% from the 10–90% quantiles, and the corresponding estimation coefficients of digital economic variables can be obtained, as shown in Table 3. Taking the quantile of GTFP as the X axis and the estimated coefficient of digital economic variables as the Y axis, a smooth curve of the estimated coefficient of digital economy to GTFP can be obtained as shown in Figure 1.

With the increasing quantile of GTFP, the coefficient of the influence of digital economy on GTFP gradually increases, ranging from 0.2678 to 0.5929. Specifically, the coefficients at the 0.1, 0.15, 0.2, 0.25, 0.3, 0.35, 0.4, 0.45, 0.5, 0.55, 0.6, 0.65, 0.7, 0.75, 0.8, 0.85 and 0.9 quantiles are: 0.2678, 0.2866, 0.3056, 0.3225, 0.3387, 0.3550, 0.3748, 0.3960, 0.4138, 0.4384, 0.4583, 0.4776, 0.4954, 0.5138, 0.5336, 0.5569 and 0.5929, showing a gradual upward trend. Except for the 0.1 and 0.15 quantiles, the digital economic coefficients of other quantiles are significant at least at the 10% level. This shows that the digital economy has a clear effect on the promotion of a city’s GTFP, and there are significant differences among cities. The higher the GTFP of a city, the greater the role of digital economy in promoting the city’s GTFP.

The influence characteristic of digital economy on the conditional distribution of city’s GTFP can be explained as follows. The first is that the digital economy is a knowledge-based economy. The digital industry is used to gather digital ecology, and digital talents are used to boost the digital industry and gradually release its potential, thereby, increasing city’s GTFP. Most of the cities with high GTFP are economically developed and are more attractive to digital talents. Compared with cities with low GTFP, the digital talent pool is more abundant, the potential of the digital industry is better released, and the industrial structure is optimized and upgraded, thereby, effectively improving the city’s GTFP. 

The second is that the carrier of the digital economy is the information network. Cities with higher GTFP have a higher level of digital economy development, and the level of industrial digitization and digital technology application is more advanced, which has a more significant incentive effect on city innovation to, thus, promote the green, coordinated and efficient development of a city’s economy. 

In recent years, some economically developed cities in China have established big data trading platforms relying on digital technology, optimizing the allocation mode of innovation elements. The effective input of city innovation elements can improve the innovation ability, stimulate the endogenous development momentum of cities. Take Wuhan as an example. In 2015, Wuhan established five major trading platforms, including the Donghu District Big Data Trading Center, which not only awakened a large number of “sleeping” data but also promoted the circulation and trading of various innovative elements and then cultivated and strengthened new kinetic energy of city economic development.

### 5.3. Dynamic Impact of Digital Economy on GTFP

Due to differences in resource endowments and development stages between cities at different levels in China, the influence of digital economy on city’s GTFP may be heterogeneous at the city level; therefore, it is necessary to discuss it deeply. According to relevant government documents (according to the “National New-type Urbanization Plan (2014–2020)”), this paper classifies municipality directly under the central government, provincial capital, municipality with independent planning status as central cities and other prefecture-level cities as peripheral cities. This paper further uses the PVAR model to analyze the dynamic impact of the digital economy on the GTFP of the whole country, central cities and peripheral cities.

The prerequisite for the establishment of the PVAR model is that the panel data is stable. First, the panel data is tested for stationarity to avoid the pseudo-regression problem caused by the estimation of non-stationary variables. This paper comprehensively uses two representative test methods: LLC test in the same root test and ADF-Fisher test in different root tests. The results show that the variables have passed the stationarity test, and the PVAR model can be established. In order to avoid the estimation error caused by the time effect and individual effect in the model, the “intra-group mean difference method” is first used to eliminate the time effect—that is, the time effect is first removed by subtracting the group mean from each variable. 

Then, we use the “forward mean difference method” (also known as the Helmert process) to eliminate individual effects. For the selection of the lag order, comprehensively considering the AIC, BIC and HQIC criteria and combined with the convergence trend of the impulse response graph, it is determined that the optimal lag order of the whole country, central cities, and peripheral cities are all 2. Then, the PVAR model was estimated.

**Figure 2:**
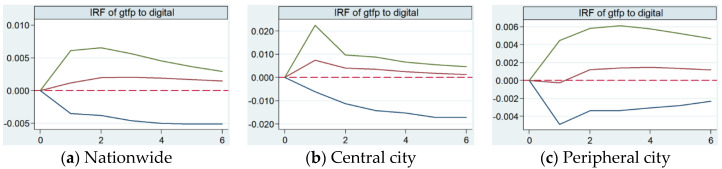
The impulse response diagram of GTFP faced with the shock of digital economy. (The green and blue lines represent the upper and lower boundaries of the 95% confidence interval respectively, and the red lines in the middle are the impulse response trace).

The impulse response function characterizes the dynamic interaction effects and the time delay relationship between variables. With the help of Stata16 software, through Monte Carlo simulation 500 times, the influence of the development of the digital economy in the whole country, central cities and peripheral cities on the current and future values of GTFP were analyzed. The impulse response estimation results of the PVAR model are shown in Table 4, and the impulse response diagram is shown in Figure 2. 

The horizontal axis is the number of impulse response periods, which is set to six periods, and the vertical axis is the response degree of GTFP faced with the shock of digital economy. The following conclusions can be drawn from Table 4 and Figure 2.

(1) The response of GTFP in national and central cities to the impact of digital economy shows positive promotion, and the response curve first rises and then falls and gradually converges to zero. The response of peripheral cities is first suppressed and then promoted, the cumulative effect is positive, and the response curve finally converges to zero. This shows that the digital economy has indeed played a positive role in promoting China’s GTFP. In recent years, the Chinese government has launched a series of forward-looking digital infrastructure construction policies, especially the comprehensive implementation of the network power strategy and the national big data strategy, which has successfully transformed China’s super-large market and demographic dividends into data dividends. Through data circulation, cooperation and sharing, the upstream and downstream blockages of the supply chain are opened up, resource allocation is optimized, and GTFP is improved.
(2) In the face of an orthogonal shock of the digital economy, the response intensity of GTFP in the whole country, central cities and peripheral cities decreases successively, with the response peak values of 0.0021 in the third period, 0.0075 in the first period and 0.0015 in the fourth period, respectively. In addition, the peripheral cities have a negative response in the early stage. Central cities have the fastest response speed and the greatest response intensity, which shows that the digital economy has the best effect on promoting GTFP of central cities. The response speed of peripheral cities is slower than the national average, and the response intensity is also lower than the national average. The digital economy has a negative impact on the GTFP of peripheral cities in the early stage, and this gradually turns to a positive impact after the first stage. This shows that there is still much room for digital economy in peripheral cities to improve GTFP. Mitrovic (2020) stated that digital information has the characteristics of spillover and sharing, it is easier to catch up with digital informatization [80]. Therefore, peripheral cities should firmly grasp the development opportunities of digital economy, strive to narrow the digital divide and fully stimulate the role of digital economy in promoting city’s GTFP.
(3) The cumulative effects of GTFP in the whole country, central cities and peripheral cities facing the shock of digital economy are 0.0104, 0.0207 and 0.0063, respectively. The cumulative effect of central cities is the largest, while that of peripheral cities is lower than the national average. This shows that, from the dynamic perspective of long period span, the digital economy has the greatest effect on the improvement of GTFP in central cities and the smallest effect on the improvement of GTFP in peripheral cities. The digital economic dividend presents a distribution pattern with more central cities and fewer peripheral cities. Compared with central cities, the digital infrastructure of peripheral cities is weaker, the development of digital industry still lags far behind that of central cities, and the integration level of digital economy and traditional industry is also lower than that of central cities. Peripheral cities should learn from the development experience of digital economy in central cities and combine their own comparative advantages to firmly seize the important opportunity of the digital economy and strive to overtake in corners.

### 5.4. Robustness Test

Through the above empirical analysis, we proved that the digital economy had a significant positive impact on the GTFP of Chinese cities. Moreover, there is heterogeneity of the influence effects in different quantiles of GTFP; in eastern, central and western regions; and between central cities and peripheral cities of China. However, this study may also have endogenous problems due to omitted variables and bidirectional causality. On the one hand, although this paper comprehensively considered various factors affecting GTFP, there are still some factors that are difficult to characterize and measure, such as institutional differences and cultural differences between regions. 

On the other hand, a city’s GTFP may, in turn, affect the development level of a city’s digital economy. The level of technological innovation is an important driving force for the development of the digital economy, and GTFP represents the level of technological innovation to a certain extent. Therefore, in order to ensure the reliability of the research results, this paper adopts the instrumental variable Tobit (IV-Tobit) method to alleviate the endogeneity problem.

(1) IV-Tobit method. Considering the fact that digital economy takes information network as an important carrier, this paper draws on the research of Huang Qunhui et al. (2019) [81] and selects the number of fixed telephones per million people in each city in 1984 as an instrumental variable for the development level of the city’s digital economy. 

On the one hand, as far as China’s internet access technology is concerned, it initially starts with telephone line dialing (PSTN). The internet is the continuation and development of traditional communication technology. The basis of telecommunications in history will influence the popularization and application of subsequent internet technologies from factors, such as technical level and usage habits and meet the assumptions of the relevance of instrumental variables. 

On the other hand, the influence of fixed telephone, a traditional communication tool, on economic development is gradually diminishing. The number of fixed-line telephones in history will not have an impact on the city’s current GTFP, which satisfies the assumption of the exclusivity of instrumental variables. In view of the fact that the original data of the selected instrumental variable is cross-sectional data, they cannot be used for quantitative analysis of panel data in this paper. 

Referring to the processing method of Nunn and Qian (2014) [82], the time-varying variable of the number of internet users in the previous year is introduced to construct the interactive term between the number of fixed telephones per 10,000 people in each city in 1984 and the number of internet users in the previous year as a tool variable. The regression results of IV-Tobit are shown in Table 5. The first stage regression results are listed in column (1), and the second stage regression results are listed in column (2). 

The regression results of the first stage show that the coefficient of the instrumental variable is significantly positive at the 1% level, indicating that there is a significant positive correlation between city’s traditional communication tools and the development level of the digital economy. The regression results of the second stage show that, although the coefficients estimated by the instrumental variable method fluctuate compared with the benchmark regression, the sign and significance of the core variable are unchanged; therefore, the benchmark regression results are relatively robust.

Considering the influence of the validity of instrumental variables on the estimation results, this study uses Kleibergen-Paap rk LM statistics to test whether the instrumental variables are related to the endogenous explanatory variables, which represent the digital economy. The results reject the null hypothesis of “insufficient identification of instrumental variables” at the 1% level, indicating that there is a strong correlation between instrumental variables and endogenous variables. 

For the test of weak instrumental variables, the Kleibergen-Paap rk Wald F statistic is 113.960, which is larger than the critical value of Stock-Yogo test at 10% level that is 16.38, and the Cragg-Donald Wald F statistic is 152.137, which is also larger than the critical value of Stock-Yogo test at the 10% level. All of them reject the null hypothesis of weak identification of instrumental variables, and it can be considered that there is no weak instrumental variable problem. Overall, the above test results illustrate the rationality of the instrumental variables selected in this paper.

(2) A Two-way fixed effect model is used to test the robustness. The empirical test is carried out by using the two-way fixed effect model including individual and time, and the regression results are shown in Table 6. The first column contains no control variables, and the second column contains all control variables. The results show that, compared with the Tobit benchmark regression, the regression coefficients of the digital economy estimated by the two-way fixed effect model are similar in size and consistent in significance, which indicates that the benchmark regression results are relatively robust.

### 5.5. Influence Mechanism Test

Through the previous theoretical analysis, we concluded that the digital economy promotes the improvement of GTFP by optimizing and upgrading the industrial structure. The following empirical tests are conducted on this mechanism through the mediation effect model, and the Tobit regression results are shown in Table 7. On the basis of model (1) proving that digital economy has a positive impact on GTFP, model (2) tests whether digital economy promotes the optimization and upgrading of city’s industrial structure. 

The regression coefficients of digital economy variable in the above two models are both positive and significant at the level of 1%. Finally, the intermediary variable of industrial structure is put into the regression equation of the influence of digital economy on GTFP, and we found that the regression coefficient of industrial structure on a city’s GTFP in model (3) is positive and significant at the level of 1%. At the same time, the influence coefficient of digital economy on city’s GTFP in model (3) is lower than that in model (1). Through the significance of the above core explanatory variable and the change of coefficient value, we verified that the upgrading of industrial structure is the mechanism of digital economy to improve the GTFP.

In order to ensure the accuracy of the conclusions, the Sobel test method was used at the same time, and the Bootstrap method was used to further test the mediation effect. The Sobel test shows that the Sobel Z value is 2.945, P = 0.003, and the mediating effect accounts for 20.0%. The Bootstrap mediation effect test was further carried out. The test results are shown in Table 8. The 95% confidence interval of the mediation effect does not contain 0, that is, the mediation effect is significant. Based on the above-mentioned intermediary effect test, the transmission mechanism of digital economy development to improve city’s GTFP by optimizing and upgrading city industrial structure exists.

## 6. Discussion and Policy Recommendations

Vigorously developing the digital economy, releasing the dividends of industrial structure transformation and creating a new engine for economic development are important issues for China’s economy to achieve green transformation and sustainable development. This paper studies the influence of digital economy on the development of city’s green economy through the Tobit model, quantile regression model and impulse response function, and we focused on the intermediary mechanism of industrial structure by using the intermediary effect model and drew the following conclusions.

(1) The influence coefficients of the digital economy on the GTFP of the whole country, the eastern and central region and the western region are 0.3549, 0.4194 and −0.1495, respectively. This shows that the digital economy can significantly promote China’s GTFP. China has gradually explored a digital economy development path that is suitable for the development environment of emerging markets and different from western developed countries. The digital economy has become an important engine to promote the high-quality development of China’s economy. However, there are clear regional differences. The digital economy in the eastern and central regions has a good effect on promoting the GTFP, while the role of digital economy in promoting GTFP in the western region has not yet appeared.
(2) The influence characteristic of the digital economy on the conditional distribution of city’s GTFP is shown as follows: with the increase of the quantile of GTFP, the influence coefficient of digital economy on it gradually increases, with the value ranging from 0.2678 to 0.5929. This shows that the digital economy has a clear effect on the promotion of city’s GTFP, and there are significant differences among cities. The higher the GTFP, the greater the promotion effect of the digital economy on the city’s GTFP.
(3) The results of dynamic influence of the digital economy on GTFP are as follows: From a dynamic long-term perspective, the digital economy has indeed positively promoted China’s GTFP. The digital economy had the best effect on promoting GTFP in central cities. There is still much room for the digital economy of peripheral cities to improve the GTFP. The digital economic dividend presents a distribution pattern with more central cities and fewer peripheral cities.
(4) Through the Stepwise causality method of intermediary effect, Sobel test and bootstrap intermediary effect test, we verified that digital economy can improve a city’s GTFP by optimizing and upgrading industrial structure. The digital economy can accelerate the transformation of new and old kinetic energy by empowering the transformation and upgrading of traditional industries and promote the green development of the economy.
  At the same time, the digital economy facilitates the transformation of the organizational form of the manufacturing industry chain and reshapes the value distribution form of the manufacturing industry chain. The rise of the digital economy platform has opened up new market space for the industrialization of digital technology, spawned a number of new industries and new business forms, injecting vitality into economic development and promoting the economy to take a path of sustainable development.

The policy implications of this paper are as follows.

(1) China should seize the opportunity of digital economy development, build new competitive advantages of the country and promote GTFP through digital economy development so as to realize green economic development. It is necessary to promote the deep integration of digital technology and the real economy, fully tap the huge potential of data as a production factor, unswervingly build digital China, strengthen key core technology research, give full play to the advantages of China’s new nationwide system and super-large-scale market, grasp the autonomy of developing digital economy, avoid the “bottleneck” problem, accelerate the construction of digital infrastructure, open up the information “main artery” of economic and social development, promote the rapid flow of various resource elements and enhance the resilience of China’s economic development.
(2) In the process of promoting GTFP through the digital economy, China should pay attention to the important mechanism of industrial structure upgrading through vigorously developing the digital economy, assisting the transformation and upgrading of traditional industries, eliminating outdated production capacity and promoting the iterative upgrade of new and old kinetic energy. The digital economy helps China to walk a sustainable development path, improve the green development level of China’s economy and lay a solid foundation for China to realize peak carbon dioxide emissions by 2030 and carbon neutrality by 2060.
  Efforts should be made to promote the digitalization of manufacturing, service and agriculture industries as well as to use digital technology to comprehensively transform traditional industries, reduce energy consumption, increase GTFP, promote the development of digital industry in key areas, improve the competitiveness of key links in the industrial chain, unblock the upstream and downstream blocking points in the industrial chain, improve the GTFP and gradually explore a digital economy development path that suits the development environment of emerging markets.
(3) Efforts should be made to narrow the gap between the western region and the eastern and central regions as well as between peripheral cities and central cities in terms of digital economy promoting GTFP. China needs to make overall plans for digital infrastructure represented by technologies, such as the internet, big data, cloud computing and artificial intelligence; intensify the construction of digital infrastructure in relatively backward areas; and strive to narrow the gap of digital infrastructure among regions. Late-developing regions should seize the important opportunity of the digital economy to narrow the gap with developed regions.
  It is necessary to optimize the regional layout and achieve differentiated positioning for different regions, give full play to the comparative advantages of the regions and realize the complementary advantages of the regions. It is necessary to make full use of the radiating and leading role of central cities, extend the digital industry chain to peripheral cities, strengthen the industrial cooperation between cities, build digital economy demonstration cities, promote cities with a high level of digital economy development and backward cities to build digital economy platforms together, realize the cross-city and barrier-free flow of data elements and smooth the circulation of domestic data elements.

## Figures and Tables

**Table 1:** Descriptive statistics.

Variables Type	Variables	Mean	Standard Deviation	Minimum Value	Maximum Value
Explained variable	Green total factor productivity (GTFP)	0.7239	0.1219	0.3584	1.4050
Explanatory variable	Digital economy (digital)	0.0511	0.0490	0.0067	0.5568
Mediating variable	Industrial structure (indus)	2.0149	0.0464	1.8574	2.1788
	Environmental regulation (env)	0.0035	0.0015	0	0.0123
	Innovation and entrepreneurship (ie)	3.7627	0.7610	0.8609	4.6151
	Marketization level (mar)	0.7452	0.2991	0.0506	2.8982
Control variables	Foreign direct investment (fdi)	0.0163	0.0171	0	0.1907
	Human capital investment (hci)	0.1632	0.0340	0.0000	0.3047
	Financial development (fin)	0.6518	0.2485	0.1115	2.3629
	Government financial expenditure on science and technology (gov)	0.0161	0.0159	0.0006	0.1880
	Number of companies (com)	6.5667	1.1025	0	9.3096

**Table 2:** Regression results.

Variables	Nationwide		East and Central		West	
	Tobit	OLS	Tobit	OLS	Tobit	OLS
Digital	0.3549 *** (0.0755)	0.2497 *** (0.0595)	0.4194 *** (0.0767)	0.2940 *** (0.0593)	−0.1495 (0.2566)	0.1955 (0.2540)
Control variable	Yes	Yes	Yes	Yes	Yes	Yes
_cons	0.7126 *** (0.0323)	0.6338 *** (0.0206)	0.7709 *** (0.0381)	0.7195 *** (0.0241)	0.6039 *** (0.0681)	0.4392 *** (0.0448)
Observations	2574	2574	1809	1809	765	765

Note: *** indicates significant at the level of 1%. The system standard errors are in parentheses.

**Table 3:** Quantile regression results.

Quantile	0.1	0.25	0.5	0.75	0.9
Digital	0.2678 (0.2161)	0.3225 * (0.1655)	0.4138 *** (0.1388)	0.5138 ** (0.2120)	0.5929 ** (0.3016)
Control variables	Yes	Yes	Yes	Yes	Yes
Observations	2574	2574	2574	2574	2574

Note: *, ** and *** indicate significant at the level of 10%, 5% and 1%, respectively. The system standard errors are in parentheses.

**Table 4:** Impulse response estimation results.

Regions	Variables	Response Intensity	Responding Speed	Cumulative Effect
Nationwide	digital→GTFP	0.0021	3	0.0104
Central city	digital→GTFP	0.0075	1	0.0207
Peripheral city	digital→GTFP	0.0015	4	0.0063

Note: The left side of the arrow is the variable that produces the shock, and the right side is the variable that responds to the shock. The response intensity represents the response peak, and the larger the absolute value, the greater the response intensity. The response speed is the time to reach the peak value. The smaller the value, the faster the response. The cumulative effect represents the sum of the impulse response values during the impulse response period.

**Table 5:** Regression results of IV-Tobit.

Variables	(1)	(2)
Digital		0.7113 *** (0.2539)
Tele	0.0118 *** (0.0010)	
Control variables	Yes	Yes
_cons	−0.0609 *** (0.0071)	0.6462 *** (0.0218)
Kleibergen-Paap rk LM Statistic		95.217 [0.000]
Kleibergen-Paap rk Wald F Statistic		113.960 {16.38}
Cragg-Donald Wald F Statistic		152.137 {16.38}
Observations	2574	2574

Note: *** indicates significant at the level of 1%. The system standard error is within (). The *p* value is within []. The critical value at the level of 10% of the Stock-Yogo weak recognition test is within {}.

**Table 6:** Two-way fixed effect regression results.

Variables	(1)	(2)
Digital	0.3327 *** (0.0938)	0.3049 *** (0.0938)
Control variable	No	Yes
_cons	0.6932 *** (0.0347)	1.2278 *** (0.0787)
City fixed effect	Yes	Yes
Year fixed effect	Yes	Yes
Observations	2574	2574
$R^{2}$	0.4922	0.5125

Note: *** indicates significant at the level of 1%. The system standard errors are in parentheses.

**Table 7:** Intermediary mechanism test of industrial structure.

Variables	GTFP	Indus	GTFP
	(1)	(2)	(3)
Digital	0.3549 *** (0.0755)	0.1399 *** (0.0133)	0.3204 *** (0.0776)
Indus			0.1933 * (0.1032)
Control variables	Yes	Yes	Yes
_cons	0.7126 *** (0.0323)	1.9161 *** (0.0077)	0.3463 * (0.1980)
Observations	2574	2574	2574

Note: * and *** indicate significant at the level of 10% and 1%, respectively. The system standard errors are in parentheses.

**Table 8:** Bootstrap mediation effect test.

	Observed Coef.	Z	P > |z|	Normal-Based [95% Conf. Interval]	
ind_eff	0.050	3.14	0.002	0.019	0.081
dir_eff	0.200	3.95	0.000	0.100	0.299

## Data Availability

Not applicable.
